# Role of Mitochondria in Cancer Immune Evasion and Potential Therapeutic Approaches

**DOI:** 10.3389/fimmu.2020.573326

**Published:** 2020-10-16

**Authors:** Katherine Klein, Kewen He, Ahmed I. Younes, Hampartsoum B. Barsoumian, Dawei Chen, Tugce Ozgen, Sara Mosaffa, Roshal R. Patel, Meidi Gu, Jose Novaes, Aarthi Narayanan, Maria Angelica Cortez, James W. Welsh

**Affiliations:** ^1^Department of Radiation Oncology, The University of Texas MD Anderson Cancer Center, Houston, TX, United States; ^2^McGovern Medical School at UTHealth, Houston, TX, United States; ^3^Department of Radiation Oncology, Shandong Cancer Hospital Affiliated to Shandong University, Jinan, China; ^4^Ankara University Faculty of Medicine, Ankara, Turkey; ^5^Department of Molecular Biosciences, The University of Texas at Austin, Houston, TX, United States; ^6^Department of Internal Medicine, Jacobi Medical Center/Albert Einstein College of Medicine, The Bronx, NY, United States

**Keywords:** mitochondria, cancer, immune function, metabolism, immunotherapy, radiation therapy

## Abstract

The role of mitochondria in cancer formation and progression has been studied extensively, but much remains to be understood about this complex relationship. Mitochondria regulate many processes that are known to be altered in cancer cells, from metabolism to oxidative stress to apoptosis. Here, we review the evolving understanding of the role of mitochondria in cancer cells, and highlight key evidence supporting the role of mitochondria in cancer immune evasion and the effects of mitochondria-targeted antitumor therapy. Also considered is how knowledge of the role of mitochondria in cancer can be used to design and improve cancer therapies, particularly immunotherapy and radiation therapy. We further offer critical insights into the mechanisms by which mitochondria influence tumor immune responses, not only in cancer cells but also in immune cells. Given the central role of mitochondria in the complex interactions between cancer and the immune system, high priority should be placed on developing rational strategies to address mitochondria as potential targets in future preclinical and clinical studies. We believe that targeting mitochondria may provide additional opportunities in the development of novel antitumor therapeutics.

## Introduction

As much of cells’ metabolic signaling takes place in mitochondria, or is regulated by mitochondrial activities, knowledge of mitochondrial function is critical to the discussion of cancer cell metabolism. In the 1920s, Otto Warburg observed that cancer cells have much higher rates of glycolysis than their differentiated cell counterparts (1). He called this phenomenon “aerobic glycolysis,” because cancer cells tend to rely on glycolysis even under normoxic conditions, a condition later called the “Warburg effect.” At the time, Warburg hypothesized that the shift to aerobic glycolysis resulted from dysfunctional mitochondria, and that this metabolic shift was a cause of cancer. However, having functional mitochondria was later found to be essential in several types of cancer, prompting the search for alternative hypotheses. Some studies suggest that eliminating cancer cell mitochondrial DNAs reduces the growth rates and tumorigenicity of those cells (2–4). Perhaps, a more likely explanation for the Warburg effect (i.e., aerobic glycolysis) is that because cancer cells are highly proliferative, their metabolic needs are different from those of differentiated cells, most of which rely on oxidative phosphorylation (OXPHOS) for energy production in the form of adenosine triphosphate (ATP). Aerobic glycolysis is less efficient for producing ATP but is beneficial to rapidly proliferating cells in terms of the formation of other required metabolites such as nucleotides, amino acids, and lipids, that is, biomass precursors (5). Moreover, even though cancer cells have high rates of glucose consumption *via* glycolysis, a significant proportion of their ATP is still produced *via* OXPHOS, again suggesting that at least some mitochondrial function is preserved in cancer (6, 7).

In addition to the requirement for functional mitochondria, cancer cells also have other characteristics that can contribute to tumor growth and progression. One example is the production of reactive oxygen species (ROS), which are produced in larger quantities by cancer cells relative to differentiated cells (8). Increased ROS may lead to increases in mutations, particularly in the mitochondrial DNA (mtDNA) owing to the close proximity of mitochondrial ROS and mtDNA and the latter’s limited capacity for DNA “proofreading” (9). These mutations can lead to oncogenic transformation and promote cancer progression. The mitochondrial genome contains mainly genes encoding for electron transport chain proteins, and mutations in these genes can therefore affect electron transport chain signaling pathways. This can then create an endless cycle in which mutated electron transport chain proteins cause increased leakage of electrons and more ROS production, which can lead to more mutations (8). Some evidence has also shown that ROS can themselves act as signaling molecules that promote oncogenic pathways, which may explain why cancer cells display increased ROS production (10). This concept is supported by the observation that knockout of genes involved in mitophagy (a mechanism by which damaged or excess mitochondria are selectively eliminated) increases intracellular ROS generated from damaged mitochondria, leading to oncogenesis (11).

In the sections that follow, we first discuss the biological rationale for mitochondria-targeted antitumor therapy, including the important relevance of mitochondrial characteristics in cancer progression and in how cancer evades the immune response. We then describe several potential mitochondria-focused antitumor therapeutic approaches.

## Crosstalk Between Hypoxia-Induced Signaling and Mitochondria

As noted previously, malignant cells rely on functional mitochondria for survival, proliferation, and metastasis. A key characteristic of tumor microenvironments that also affects cellular survival, proliferation, and metastasis is hypoxia and its consequent effects on cellular signaling pathways. Below we provide some examples of how signaling pathways induced by hypoxia regulate energy metabolites and mitochondrial biogenesis.

Aberrant signaling pathways in cancer can allow malignant cells to adapt to the hypoxic environment. The ability to detect and adapt to fluctuations in cellular oxygen levels relies heavily on hypoxia-inducible factor (HIF) and its two components: HIF-1α and HIF-1β [the latter also known as aryl hydrocarbon receptor nuclear translocator (ARNT)]. Only HIF-1α can sense oxygen directly; it is stable under hypoxic conditions (12) but under low-oxygen conditions it upregulates several genes to promote cancer cell survival, as described below.

Under hypoxic conditions, HIF-1α first activates *PDK1* (pyruvate dehydrogenase kinase 1) (13). Although the HIF1α-PDK1 axis generally stimulates glycolysis (14), under hypoxic conditions increased glycolysis is needed to produce energy when the low oxygen levels cannot support OXPHOS. Perhaps, not surprisingly, this axis also influences mitochondrial function. PDK1 phosphorylates and inhibits pyruvate dehydrogenase, which results in a shortage of pyruvate to support the tricarboxylic acid (TCA) cycle. This shortage causes a drop in mitochondrial oxygen consumption and leads to a relative increase in intracellular oxygen tension, which promotes cell survival under hypoxic conditions (15). The catalysis of pyruvate to lactate by lactate dehydrogenase A is a key checkpoint in anaerobic glycolysis. Hypoxia induces the expression and activation of lactate dehydrogenase A *via* HIF-1α in tumor cells (16), which in turn enhances the conversion of pyruvate to lactate and suppresses both the TCA cycle and oxygen consumption. Collectively, these effects lead to the production of metabolites for mitochondrial biogenesis, which is also beneficial for cancer cell survival in hypoxic environments. Given the importance of HIF-1α in regulating mitochondrial function, regulation of the activity of HIF-1α is also critical. Perhaps, the best-known regulator of HIF-1α is prolyl hydroxylase. In hypoxic conditions, the activity of prolyl hydroxylase is decreased, which stabilizes HIF-1α (17) and upregulates PDK1, which in turn inhibits prolyl hydroxylase activity (13). HIF-1α can also be regulated independently of hypoxia by pathways related to growth factors (18) and Ras/Raf/MEK (19).

In addition to HIF-1α, another promising target for antitumor therapy is peroxisome proliferator-activated receptor γ coactivator-1 alpha (PGC-1α). A potent transcriptional coactivator, PGC-1α has been proposed to coactivate HIF1α during hypoxia. PGC-1α is a positive regulator of mitochondrial biogenesis and increases the expression of numerous ROS-detoxifying enzymes, thereby acting as a powerful regulator of ROS removal (20).

Another fundamental stress condition, that of hypoxia-reoxygenation, is also known to impair mitochondrial function. Reoxygenation results in accumulation of ROS, which induces the overexpression of dynamin-related protein 1 (Drp1), a mitochondrial fission-accelerating factor that is regulated by a series of signaling pathways including AMPK (21) and Ras-Raf-ERK (22). Mitochondrial fission is a process by which long, tubular mitochondria are separated into two or more parts, thereby increasing the number of mitochondria. Hypoxia-induced Drp1 overexpression and mitochondrial fission have been linked with increased tumor-cell migration and metastatic activity (23). Mitochondrial fission also requires stabilization of HIF-1α (24). Therefore, identification of crosstalk between HIF-1α and Drp1 could provide novel insights for developing cancer therapies.

## The Role of Mitochondria in T-Cell and Macrophage Differentiation and Function

Mitochondria also supply the ATP necessary for the rapid proliferation, differentiation, and effector function of T cells, which are major components of antitumor immunity (25, 26). Each phenotypic stage of T cells has specific metabolic demands and signaling pathways that facilitate their respective functions. For example, naïve T cells are quiescent, relying on OXPHOS to maintain energy demand. However, upon their activation through encounters with T-cell receptors and costimulatory signals, the metabolism of these T cells shifts to glycolysis to support their rapid growth and production of biosynthetic factors for differentiation into effector T cells. Although aerobic glycolysis is less efficient than OXPHOS for yielding ATP, it generates metabolic intermediates that are important for cell growth and proliferation as well as for cytotoxicity and cytokine production. Several mitochondrial energy metabolism pathways have been implicated in shaping T-cell function and differentiation. The central network underlying this reprogramming is the PI3K/AKT/mTOR pathway, which boosts glycolytic activity in T cells *via* activation of transcription factors such as HIF-1α and Myc (27). On the other hand, regulatory T cells (Treg) and memory T cells rely on OXPHOS and fatty acid oxidation (FAO) to support their survival and differentiation (28). After antigens are cleared, a small fraction of T cells participates in the transition from effector to memory state *via* enforcing FAO by elevating AMPK activity or by inhibiting mTOR (29). Mitochondrial ROS which function as signaling intermediates are responsible for activating T cells and promoting antigen-specific proliferation (26). A balance in the ROS levels helps T-cell proliferation, activation and apoptosis (30, 31). In light of this, means of modulating ROS levels may be important for prolonging the survival of T cells and improving their antitumor function.

T-cell reprogramming is also associated with changes in mitochondrial structures. In normal cells, activation of mitochondrial respiration leads to increased fusion of the mitochondria, with subsequent expanded cristae space (32). With regard to T cells in particular, naive T cells have fragmented and round mitochondria and use OXPHOS for energy generation. Upon activation *via* T cell receptors, T cells undergo a metabolic shift to aerobic glycolysis, in which mitochondrial fission increases, leading to increased numbers of mitochondria and loosened cristae (33). This loosening of the cristae stretches out the components of the electron transport chain, leading to less efficient electron transfer, inefficient OXPHOS, and increased aerobic glycolysis, as well as increased ROS generation, which resembles the increase in ROS noted in cancer cells. In the conversion from an effector T cell to a memory T cell, mitochondria become increasingly fused, with tighter cristae, expanded space, and more efficient OXPHOS (33). Memory T cells thus rely more on OXPHOS than on aerobic glycolysis and have a greater mitochondrial respiratory capacity than effector T cells (34). This primes memory T cells for longevity. Although effector T cells seem to rely less on mitochondrial pathways of cellular respiration (relative to memory T cells), mitochondrial integrity is still critical for effector T-cell function. In one model of clear cell renal cell carcinoma, effector T cells were found to be impaired, with small, fragmented mitochondria and abundant ROS; activation of the effector T cells in this model was improved by addition of pyruvate or mitochondrial ROS scavengers, which also served to improve mitochondrial function (35).

Another immune cell worth mentioning, in which mitochondria plays a vital role in cells’ differentiation and activity, is the macrophage. There are two main subtypes of macrophages that each have distinct metabolism phenotypes. The polarization of pro-inflammatory macrophage (M1 subtype), which is activated by lipopolysaccharide (LPS)/IFN-γ, is believed to induce a metabolic shift from OXPHOS to aerobic glycolysis and an increase in ROS production by the mitochondria (36), while the anti-inflammatory macrophage (M2 subtype), which is activated by IL-4, is characterized by an increase in OXPHOS and FAO (37, 38). Based on these characteristic metabolic signatures, a novel anticancer therapy *via* editing macrophage polarization to pro-inflammatory M1 phenotype by chloroquine, which manipulates their metabolism shift from OXPHOS to glycolysis, inhibited tumor development (39).

On the other hand, similar to in T cells as described earlier, different phenotypes of macrophages display morphological differences in their mitochondria. In a recent study, Yue Lie and colleagues recognized this difference in mitochondrial organization through morphological analysis, which can enable researches to characterize the activation status and therefore the metabolic phenotype of macrophages. They observed that macrophages relying on glycolysis for energy production had increased mitochondrial fission, while macrophages relying on OXPHOS displayed increased mitochondrial fusion, similar to the phenomenon seen in T cells (40).

## Metabolic Competition Between T Cells and Tumor Cells in the Tumor Microenvironment

Just as cancer cells need large amounts of glucose to support their rapid proliferation, tumor-infiltrating T lymphocytes (TILs) also need glucose to support their expansion into effector T cells, thereby creating competition between cancer cells and TILs for glucose in the tumor microenvironment. As mentioned before, tumor cells ensure their own growth by reprogramming their preferred mode of energy generation to aerobic glycolysis. In this context, T cells with limited access to glucose switch to OXPHOS, which can be fueled by many nutrients but requires oxygen. As such, TILs demonstrate metabolic insufficiency that is typified by continuous loss of mitochondrial function and mass (41). One study showed that CD8+ TILs adapted to the nutrient- and oxygen-limited tumor microenvironment associated with hypoglycemia and hypoxia by enhancing PPAR-α signaling and FAO in mouse melanoma models (42). Indeed, the enhanced aerobic glycolysis of tumor cells not only depletes crucial nutrients for T cells, which dampens glycolysis, but also stimulates immune suppressive pathways, such as PD1 signaling, that enhance tumor immune evasion. Ultimately, this competition for nutrients leads to T-cell exhaustion and cancer progression.

Collectively, the aberrant metabolic activities of tumor cells lead to impaired proliferation and functioning of TILs that could be responsible for immunosuppression and immune evasion. This concept is indirectly supported by the observation that immune checkpoint blockade (e.g., anti-PDL1) can reduce the glucose consumption of tumor cells by restoring effector T-cell function (43). In addition to PD1/PDL1 and CTLA4, other “metabolic checkpoints” (e.g., mTOR, PGC-1α, IDO1, LDH) have been identified as affecting the competition between cancer and infiltrating immune cells for nutrients and metabolites (44–46). Understanding the metabolic reprogramming of tumor cells and immune cells will provide insights into potential means of regulating tumor immunity (47).

## Immune Checkpoint Inhibitors and Mitochondrial Metabolism

Immune checkpoint inhibitors like anti-PD1/PDL1 and anti-CTLA4 help to regulate the expression of immune checkpoint molecules that can be used by cancer cells to evade attacks by the immune system. Previous studies demonstrated that mitochondrial activity, which is critical for T-cell function, can be augmented by blocking PD1 or CTLA4 signaling, which consequently overcomes T-cell exhaustion (44). PD1 has been shown to affect T cells by prompting metabolic reprogramming toward depressed glycolysis and increased FAO, as well as increasing the expression of carnitine palmitoyl transferase 1A (CPT1A), which promotes the FAO of endogenous lipids (48). On the other hand, CTLA4 acts on T cells by inhibiting glycolysis with no augmentation of FAO (49). Thus, T cells that are affected by PD1 are well sustained owing to the amount of energy produced by FAO, whereas CTLA4 results in inhibition of T-cell differentiation, because naive T cells rely on glycolysis to become effector T cells. These distinct effects of CTLA4 and PD1 on the metabolic reprogramming of T cells could account for the divergent consequences of their blockade in reinvigorating exhausted T cells. In addition to restoring T-cell activity, immune checkpoints have also been shown to concomitantly depress tumor-cell metabolism. Specifically, signaling through PDL1 was found to directly upregulate glycolysis in tumor cells through activating the AKT/mTOR pathway (43), leading to enhanced glucose uptake and lactate production and the expansion and survival of these tumor cells. Conversely, a therapeutic inhibitor of PDL1 decreased the glycolysis rate *via* blocking interactions between PDL1 and the PD1 receptor, restoring glucose levels in tumor cells and limiting tumor progression (50, 51). Other immune checkpoint inhibitors, such as CD47, inhibit the phagocytosis of cancer cells by binding to the signal regulatory protein α (SIRPα) receptor expressed on macrophages and DCs (52, 53). In one study, using anti-CD47 antibodies to inhibit the CD47−SIRPα interaction activated innate immunity by promoting the destruction of cancer cells by macrophages (52). Anti-CD47 would also activate adaptive immunity by enabling the cross-presentation of engulfed antigens, mostly by DCs, leading to antitumor cytotoxic reactions, an effect that can be increased by combinatorial treatments with PD1 antibodies (54). CD47 antagonists currently being tested in clinical trials include Magrolimab (NCT03248479) and TTI 621 (NCT02890368).

Although encouraging antitumor effects have achieved by immune checkpoint inhibitors, many patients with cancer do not respond to, or less sensitive to, these drugs, probably through several mechanisms that suppress antitumor immune effectiveness under unfavorable tumor microenvironmental and metabolic conditions. Novel combinatorial strategies for such patients are therefore needed. For example, Chamoto and colleagues reported in a preclinical study (44) that ROS, by activating both AMPK and mTOR phosphorylation and by augmenting PGC-1α (a downstream target of both AMPK and mTOR known to increase mitochondrial activity), strongly activated mitochondrial function in tumor-reactive T cells and showed synergistic tumoricidal effects with PD1 blockade. They further found that direct activators of mTOR, AMPK, or PGC-1α also synergistically enhanced the antitumor effects of PD1 blockade therapy. These findings provide a proof of concept for a combinational strategy involving mitochondrial activation agents and PD1 inhibitors for patients with disease that is not responsive to PD1 inhibitors. These findings further suggest that markers of mitochondrial activation such as PGC-1α could be useful as biomarkers of the effectiveness of PD1 blockade as antitumor therapy. A direct connection between PGC-1α and PD1 was implied in another study (45) showing that PD1 could suppress both the expression and function of PGC-1α, which may explain why reintroducing PGC-1α can overcome T-cell exhaustion. Other studies have also found that increasing the FAO rate and mitochondrial respiratory capacity through mitochondrial activation by using of bezafibrate, an agonist of PGC-1a/PPAR complexes, are associated with increasing or maintaining the numbers of TILs, which ultimately leads to improved anti-PD1 responses (55). Similarly, in hypoxic and hypoglycemic tumor microenvironments (in which tumor cells and immune cells compete for nutrients), TILs promote FAO as a form of self-preservation. This in turn allows CD8+ TILs to preserve energy and their effector functions. By contrast, PD1 inhibitors slow tumor growth without interfering with the TIL function. Presumably, the synergy of increased FAO and PD1 inhibitors acts to enhance the effectiveness of immunotherapy and delay tumor growth (42).

In summary, mitochondria are strongly affected by PD1 inhibitors. In CD8+ T cells, PD1 inhibition results in metabolic reprogramming of T cells to rely on FAO, which may explain the longevity of PD1-stimulated T cells, akin to memory T cells sustaining survival *via* FOA. PD1 signaling also drastically affects the function and structure of the mitochondria. Mitochondria undergo many structural changes in response to immune checkpoint molecules, including reductions in both the number and length of mitochondrial cristae after PD1 stimulation (56). This highlights the importance of preserving mitochondrial function and structure in memory T cells.

## Targeting Oxidative Phosphorylation

The observation that glycolysis is upregulated in cancer cells relative to normal cells has led many to assume that OXPHOS is globally downregulated in cancers. However, some groups have found that a significant subset of particular cancers, such as MAPK-resistant melanoma (57), SMARCA4 mutant lung cancer (58), RB1-deficient breast cancer (59), OXPHOS-high diffuse large B-cell lymphomas (60), and oncogene-ablation−resistant pancreatic ductal adenocarcinoma (61), have increased OXPHOS, and that this upregulation can occur even in the presence of active glycolysis (62). In the OXPHOS pathway, ATP is generated by the transport of electrons to a series of transmembrane protein complexes, including complexes I, II, III, and IV, in the mitochondrial inner membrane, known as the electron transport chain. When OXPHOS is active, electrons flow through complex I, complex II, Coenzyme Q, complex III, cytochrome C, and complex IV, with oxygen acting as the terminal electron acceptor (62). The assumptions were that elevated tumor oxidative metabolism was associated with increased hypoxia in tumor regions, which forms a barrier to T cell activity, increases T-cell exhaustion, and decreases antitumor immunity; and that the lack of oxygen would cause resistance to antitumor therapy, leading to local recurrence, increased metastasis, and poor clinical outcomes. Thus, OXPHOS inhibition could theoretically reduce oxygen consumption and subsequently decrease tumor hypoxia.

Indeed, several studies have shown that OXPHOS inhibition is effective in targeting cancers in which OXPHOS is upregulated. For example, in one study treating the Ras-driven pancreatic ductal adenocarcinoma with metformin or the complex V inhibitor oligomycin significantly suppressed proliferation of tumor cells *in vitro* and retarded the growth of PDAC-215 and PDAC-A6L xenografts (61). Another study showed that non-small cell lung cancer with mutations in K-ras and deficient LKB1 are particularly sensitive to the complex I inhibitor phenformin (63). Other evidence supports the use of OXPHOS inhibitors in combination with conventional therapies such as BRAF and EGFR inhibitors, immunotherapy, or radiotherapy to overcome treatment resistance. A preclinical study found enhanced antitumor effects from combining the protein kinase BRAF inhibitor PLX4720 with the OXPHOS inhibitor phenformin in melanoma (64). Because BRAF inhibitors also induce PGC-1α, a regulator of mitochondrial biogenesis that in turn causes OXPHOS upregulation (65), combining BRAF inhibitors and phenformin could have reduced the proliferation of BRAF-mutant melanoma cells and contribute to tumor regression in that study. Similar results were reported in a phase II randomized clinical trial in which the addition of metformin (which also inhibits mitochondrial complex I) to tyrosine kinase inhibitors led to enhanced antitumor effects in epidermal growth factor receptor−mutated non-small cell lung cancer (66). Further, reliance on oxidative metabolism (as opposed to glycolysis) in tumor cells has been linked with anti-PD1 resistance (67). Indeed, that study showed that having tumor cells with high oxidative metabolism predicted poor response to anti-PD1 therapy in patients with melanoma, whereas having high glycolytic metabolism did not. Collectively, these findings suggest that OXPHOS inhibitors could be used to target cancer subtypes in which OXPHOS is upregulated and would alleviate antitumor resistance when combined with other therapies for at least some subset of tumors. Given the promising treatment efficacy of OXPHOS inhibitors, the phase 1 clinical trials of a novel OXPHOS inhibitor (IACS-010759) in leukemia (NCT02882321) and metastatic solid tumors (NCT03291938) are ongoing.

## CAR-T Cell Therapy

Another form of immunotherapy garnering significant attention is chimeric antigen receptor (CAR) T cell therapy, which involves isolating T cells from patients, engineering those cells to express different antigen-receptor molecules, and re-infusing them into patients for cancer therapy. Although CAR-T cell therapy can be quite effective against liquid tumors, its applicability to solid tumors is still under investigation. Several potential reasons for the limited effectiveness of this potentially curative therapy include the suppressive tumor microenvironment; the poor persistence of TILs after their adoptive transfer, prompting some to conclude that promoting immunologic memory in TILs may enhance antitumor immunity and the curative potential of CAR-T cell therapy for advanced cancer; and the metabolic fitness and mitochondrial functionality of these CAR-T cells.

Several studies have shown that the activity, survival, and persistence of a CAR-T cell are dictated by its transduced costimulatory domains. For example, CAR-T cells with CD28 signaling evoke enhanced glycolysis, whereas inclusion of 4-1BB in the engineered vector evokes enhanced OXPHOS and mitochondrial biogenesis (68). Signaling *via* 4-1BB also provides metabolic support to T cells by upregulating PGC1α-dependent pathways (69). Therefore, CAR-T cells with 4-1BB signaling components may evoke stronger immunotherapeutic responses that are accompanied by robust T-cell activation and increased oxidative activity. Moreover, CD28 stimulation has been shown to favor the establishment of effector memory T cells, which are particularly effective against peripheral infections owing to their cytotoxicity and location in tissues. On the other hand, 4-1BB stimulation seems to tip the balance toward long-lasting central memory T cells, which, because of their high proliferation and centralized location in lymph nodes, are more effective against systemic infections and tumor recurrence (68). The addition of cytokine-related domains to CAR-T cell constructs may be beneficial, but the nature and the function of the cytokines should be carefully considered in terms of their short-term and long-term effects. For example, interleukin (IL)-2 induces glycolysis for the initial rapid expansion of CD8+ T cells (70), whereas IL-7 and IL-15 are important in the long-term maintenance of memory T cells and increased mitochondrial biogenesis (71).

Attempts have been made to improve the effectiveness of CAR-T cell therapy by increasing the mitochondrial mass of CAR-T cells, which would drive them to produce energy under stressful conditions (72). Indeed, CAR-T cells with condensed mitochondria (which often occurs during the conversion from an effector T cell to a memory T cell) showed greater expansion capacity *ex vivo*. Boosting mitochondrial biogenesis in immune-resistant chronic lymphocytic leukemia-derived CD8+ T cells also led to significant improvement in the effectiveness of these cells (73). A mouse melanoma model reported that CD8+ TILs, under conditions of limited glucose and hypoxia, showed reduced glycolysis but enhanced FAO and that further promoting FAO enhanced the ability of CD8+ TILs to attenuate tumor progression (42). Therefore, inducing FAO in CAR-T cells could be an alternative approach to inhibit tumor progression. Another approach may involve inhibiting Akt, a protein kinase that slightly alters the glycolysis of T cells but significantly increases FAO metabolites and improves the persistence of adoptively transferred CD8+ T cells (74). Akt signaling has been shown to negatively affect mitochondrial function in TILs and to reduce PGC-1α levels. In contrast, forced expression of PGC-1α, which enhances mitochondrial biogenesis, restored the effector function of TILs despite the suppressive tumor microenvironment (41). The same concept may be useful for restoring energy and overcoming exhaustion in tumor-infiltrating CAR-T cells.

## Radiation Therapy

Radiation therapy is a critical component of many cancer treatments and understanding how mitochondria are affected by radiation is crucial for optimizing treatment and subsequent quality of life for cancer patients. Ionizing radiation is known to alter mitochondrial function, increase mitochondrial oxidative stress, and induce apoptosis, which collectively would be beneficial for killing tumor cells but is unwanted in healthy tissues (75, 76). Clinically, the main cause of radiotherapy failure is the development of cellular radioresistance, which can be conferred by various mechanisms including chances in glycolysis or mitochondrial metabolism (77); indeed, targeting those mechanisms has been shown to improve radiotherapy responses (3, 78, 79).

The mitochondrial genome is more prone to oxidative damage from ionizing radiation than is nuclear DNA because of its lack of protective histones and resultant limited repair ability (80). One group found that irradiation led to increased amounts of mtDNA that stayed elevated for up 6 months in some organs (81). Others have found that radiation increased levels of the mitochondrial biogenesis regulator PGC-1α (82), leading to further increases in mitochondrial mass and overall function (51). One study of tissue samples from patients with clear cell carcinoma linked higher mitochondrial mass with reduced tumor aggressiveness (83). Presumably, an increase in mitochondrial content could lead to subsequent strains on resources in highly proliferative cancer cells (81, 84, 85). In addition to causing increases mtDNA and PGC-1α, radiation can also inhibit mitochondrial respiration. For instance, in one preclinical study, irradiating cells led to partial deactivation of mitochondrial complexes I (32%) and III (11%), decreased succinate-driven respiratory capacity (13%), and increased ROS levels (86).

As noted earlier, tumor cells rely on glycolysis to produce energy (ATP). The hypoxic tumor microenvironment enhances radioresistance, which reduces the effectiveness of radiation therapy. Attempts to oxygenate tumors have not been successful clinically thus far. However, decreasing the tumor demand for oxygen by inhibiting mitochondrial respiration (i.e., OXPHOS) and elevating intracellular mitochondrial oxidative stress can enhance radiosensitivity (87, 88). Another potential therapeutic approach considering mitochondrial function is the combination use of an OXPHOS inhibitor and radiotherapy to overcome PD-1 resistance. Our preclinical study has demonstrated an effective combination treatment of radiation therapy (8Gy × 3 fractions) and a novel OXPHOS inhibitor (IACS-010759) in a PD-1 resistant NSCLC model (89). The combination of an OXPHOS inhibitor not only decreased tumor burden of the primary tumor (radiation-targeted tumor), but also reduced the secondary tumor (non-radiation-targeted tumor) compared to radiotherapy alone. The mechanism exploration of *in vivo* and *in vitro* studies found that radiation could induce the PD1-resistant tumor to switch from glycolysis to OXPHOS and increase immunosuppressive regulatory T cells. The addition of an OXPHOS inhibitor decreased radiation-induced regulatory T cells and increased activated CD8+ T cells.

Antioxidants, as their name implies, inhibit oxidation and thus act to prevent mtDNA damage and increase cellular resistance to oxidative stress. The primary mitochondrial antioxidant enzyme, manganese superoxide dismutase (MnSOD), has been linked with resistance and sensitivity to radiation. In one series of experiments involving human pancreatic cancer cells, overexpression of MnSOD was associated with radioresistance, and suppression of MnSOD protein levels sensitized the cells to radiation-induced cell death (90). Changes in AKT signaling (a kinase involved in proliferation and metabolism) can also confer radioresistance through prolonged exposure to radiation by activating two DNA repair pathways, non-homologous end-joining and homologous recombination (91). A recent review proposed that the electron transport chain complexes and ATP synthase respond to ionizing radiation (82). When an imbalance in the activity of electron transport chain complex/OXPHOS occurs, it can cause elevated superoxide production. However, mitochondria can respond to the radiation by increasing the mtDNA copy number. Therefore, there needs to be a delicate regulation in order to include its tumor suppressor function in cancer treatment.

Mitochondrial dysfunction has key roles in oxidative stress *via* increased ROS production, which causes mitochondrial membrane depolarization and triggers intrinsic apoptosis pathways. In one study, the Apo2/tumor necrosis factor-related apoptosis-inducing ligand TRAIL was found to specifically induce depolarization in a ROS-dependent manner in cancer cells (92). Treatments that increase mitochondrial membrane depolarization have had pro-apoptotic effects on rat pancreatic tumor cells (93). Mitochondrial permeability is regulated by permeability transition core complexes; these core complexes can be triggered by agents that increase cytosolic Ca^2+^ concentrations or stimulate ROS generation (94, 95), which can allow the entry of molecules that can lead to the swelling and rupture of the inner mitochondrial membrane, resulting in the release of cell death factors. One group found that irradiation led to decreases in the activity of mitochondrial cell-membrane Na^+^-K^+^ and Ca^2+^/Mg^2+^ ATPase and ATP content. These decreases in turn led to apoptosis *via* the intrinsic cell death pathway (96). Triggering of ROS generation can also promote sensitivity to radiation by stimulating the generation of ceramide, an important pro-apoptotic mediator that increases cytochrome c release and suppresses anti-apoptotic gene activity. Radiation-induced damage to DNA triggers the activation of mitochondrial ceramide synthase, leading to more ceramide generation and tumor cell death (97). This observation has led some to propose that a mitochondria-targeted nanoradiosensitizer could induce mitochondrial dysfunction by enabling the localized accumulation and continuous production of ROS. Several groups have explored the use of such sensitizers to activate ROS bursts and enhance radiosensitivity (98–100). In one such study, combining photon radiation with a nanosensitizer based on titanium dioxide-gold nanoparticles bearing the mitochondrial-targeting compound triphenylphosphine led to selective overproduction of ROS in the mitochondria, mitochondrial collapse, and irreversible apoptosis, which not only enhanced the antitumor effectiveness of the radiation but also led to less damage to nearby normal tissues (98).

## Conclusions

**Figure 1:**
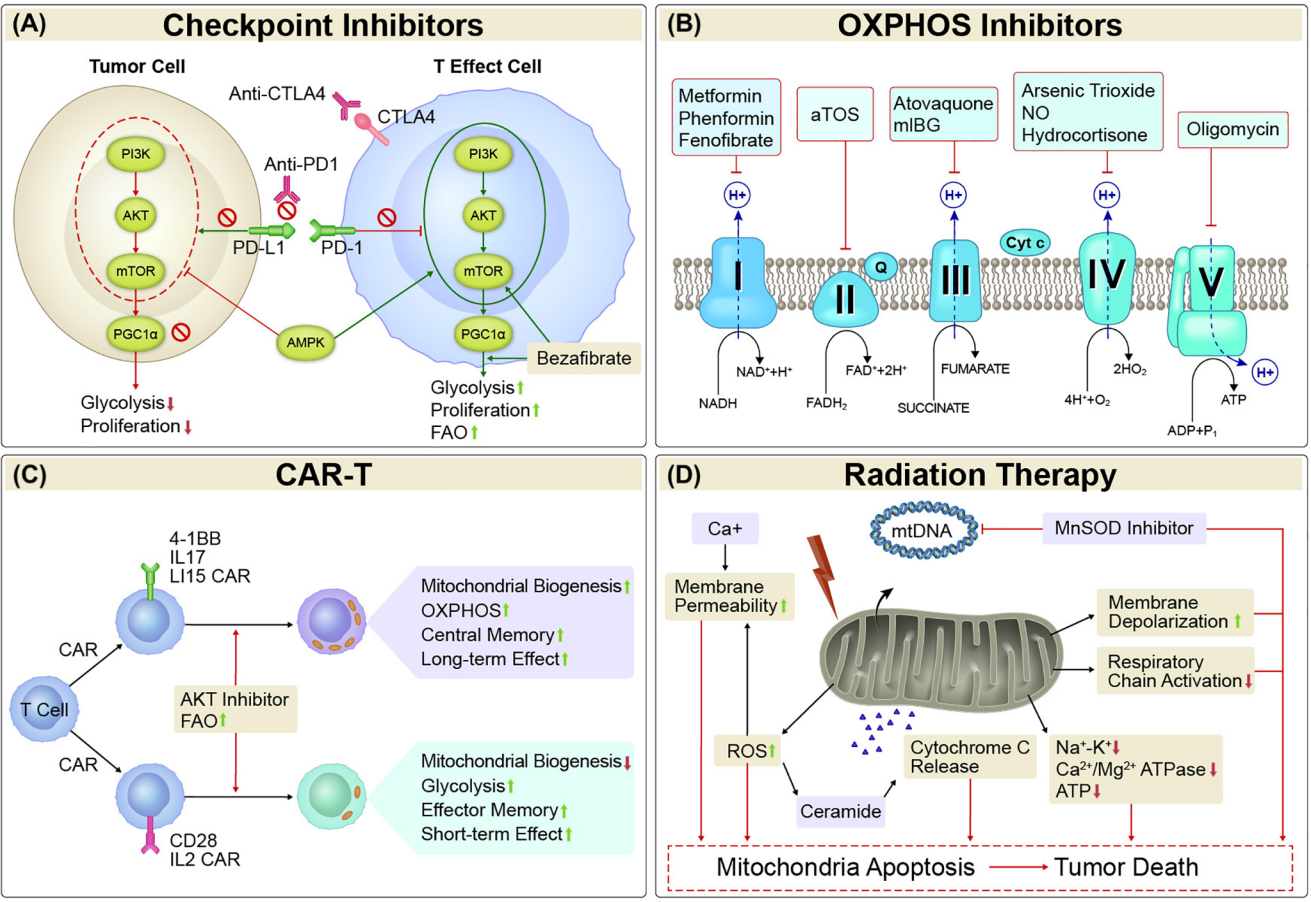
Mitochondria-targeted Therapies. **(A)** Checkpoint Inhibitors. Mitochondrial activity is strongly affected by both immune checkpoints and metabolism checkpoints. Either alone or the combination of immune checkpoint inhibitors such as PD1/PDL1 and CTLA-4 inhibitors and metabolism checkpoint inhibitors such as bezafibrate (an agonist of PGC-1α/PPAR complexes) and agents target PI3K-AKT-mTOR-PGC1α pathway could enhance glycolysis and/or FAO, and proliferation in tumor-reactive T cells, or reduce glycolysis and proliferation in tumor cells. **(B)** OXPHOS inhibitors. Cancer cells with elevated OXPHOS metabolism was associated with increased hypoxia in tumor regions, which forms a barrier to T cell activity, increases T-cell exhaustion and decreases antitumor immunity. OXPHOS inhibitors that target the transport of electrons (complexes I, II, III, IV, and V) would alleviate antitumor resistance in process of antitumor treatment in those cancers. **(C)** CAR-T. The activity, survival, and persistence of a CAR-T cell are dictated by its transduced costimulatory domains. CAR-T cells with 4-1BB, IL17, and IL15 in the engineered vector evokes enhanced mitochondrial biogenesis and OXPHOS, with central memory T cells and long-term effect, whereas inclusion of CD28 and IL2 signaling evoke decreased mitochondrial biogenesis, enhanced glycolysis and favor the establishment of effector memory T cells with a short-term effect. **(D)** Radiation Therapy. Irradiation led to increased amounts of mtDNA and mitochondrial biogenesis. It can also inhibit mitochondrial respiration. The primary mitochondrial antioxidant enzyme, MnSOD, has been linked with radioresistance. Thus, suppression of MnSOD sensitized the cells to radiation-induced cell death. Mitochondrial dysfunction has key roles in oxidative stress *via* increased ROS production, which causes mitochondrial membrane depolarization and triggers intrinsic apoptosis pathways. Treatments that increase mitochondrial membrane depolarization have had pro-apoptotic effects. Mitochondrial permeability is regulated by permeability transition core complexes, which can be triggered by agents that increase cytosolic Ca^2+^ concentrations or stimulate ROS generation, resulting in cell death. Irradiation led to decreases in the activity of mitochondrial cell-membrane Na^+^-K^+^ and Ca^2+^/Mg^2+^ ATPase and ATP content, which in turn led to apoptosis *via* the intrinsic cell death pathway. Triggering of ROS generation can also promote sensitivity to radiation by stimulating the generation of ceramide, an important pro-apoptotic mediator that increases cytochrome c release and suppresses anti-apoptotic gene activity. OXPHOS, oxidative phosphorylation; CAR-T, chimeric antigen receptor T cell therapy; MnSOD, manganese superoxide dismutase; ROS, reactive oxygen species; mtDNA, mitochondrial DNA.

Mitochondria are essential organelles with several important roles in addition to generating ATP through OXPHOS. Here, we explored the complex interactions among the tumor microenvironment, mitochondrial metabolism and biogenesis, and the immune system, with the aim of applying the knowledge gained to the development of new anticancer therapeutic approaches (**Figure 1**). One example of such therapy could involve combining metabolic therapies with immune checkpoint therapies. Such combinations could potentially increase T-cell survival and the generation of memory cells by improving energy metabolism and decreasing lymphocyte mitochondrial dysfunction and ROS generation, while at the same time controlling tumor growth by reducing the total amount of energy available in the tumor microenvironment. Another approach being tested targets OXPHOS with metformin and tyrosine kinase inhibitors, which in one study was found to extend progression-free survival among patients with non-small cell lung cancer, suggesting that targeting metabolic pathways can work synergistically with other therapies without increasing adverse events. Cellular therapy, particularly for solid tumors, may prove useful for promoting mitochondrial biogenesis and OXPHOS through the *ex vivo* mtDNA editing of CAR-T cells, which presumably would overcome the progressive loss of mitochondrial mass and PCG-1α expression characteristic of natural, un-engineered TILs. Combinations of nanoradiosensitizers and single-beam proton radiation therapy could be used to exploit mitochondrial dysfunction and create ROS “bursts” that lead to apoptotic cascades in tumors with little damage to the surrounding tissue. Future preclinical and clinical studies that address mitochondria as potential targets should be prioritized given the central role held by mitochondria in the complex interactions between cancer and the immune system.

## Author Contributions

Writing the manuscript: KK and KH. Reviewing and proofreading the manuscript: AY, HB, DC, TO, SM, RP, MG, JN, AN, MC, and JW. Supervision and oversight: MC and JW.

## Funding

Supported in part by Cancer Center Support (Core) Grant P30 CA016672 from the National Cancer Institute, National Institutes of Health, to The University of Texas MD Anderson Cancer Center.

## Conflict of Interest

The authors declare that the research was conducted in the absence of any commercial or financial relationships that could be construed as a potential conflict of interest.
